# (Dis)honorably discharged: identifying policy gaps in military–civilian reintegration

**DOI:** 10.1093/haschl/qxae021

**Published:** 2024-02-16

**Authors:** Tavis Reid, Kaitlyn M Sims

**Affiliations:** Josef Korbel School of International Studies, University of Denver, Denver, CO 80208, United States; US Army, Colorado Springs, CO, United States; Josef Korbel School of International Studies, University of Denver, Denver, CO 80208, United States

**Keywords:** US military, discharge, veteran's benefits, discipline, military culture, health care

## Abstract

Despite the substantial transition assistance available for honorably separating servicemembers, 75% of US veterans report difficulties with the transition to civilian life. For the 16% of veterans who separate with less-than-honorable discharges, these difficulties are compounded by the lack of structural support from the US military. Social stigma, limited transition programming, and loss of benefits create a perfect storm of barriers for these discharged servicemembers. These barriers compound with post-service mental and physical health challenges to contribute to cycles of misconduct that can result in criminal incarceration. Further, because most of these veterans lack health benefits from the Department of Veterans Affairs due to their discharge status, this population is substantially understudied from a public health perspective. However, actionable policy paths forward and federal policy change offer opportunity to soften the landing for these veterans and meet their legitimate needs for care.

## Introduction

The prolonged wars in Iraq and Afghanistan have created an influx of combat veterans, and military veterans now comprise 6.4% of the US population.^1^ With the conclusion of these wars come familiar, yet unresolved, challenges to servicemember reintegration into civilian life. Veterans face substantial physical, emotional, and societal barriers after exiting the service, from permanent disability to difficulty adjusting to family life. Fortunately, there many programs to help ameliorate these concerns, such as lifelong federal care through the Department of Veterans Affairs (VA), health insurance, educational benefits, home loans, and social support groups.^2,3^ While these programs are growing nationwide,^4^ service quality, availability, and timeliness vary. Furthermore, not all veterans are equally eligible, making the landscape of post-veteran reintegration uneven.

Even under the best-case scenarios, approximately 75% of servicemembers report difficulties during the transition from servicemember to civilian life.^5^ Transition difficulty contributes to the over 6000 veteran suicides annually.^6^ In 2021, the VA spent over $11 billion on mental health care and projected a 20% budgetary year-over-year growth to meet increasing service demands.^4^ However, veterans’ access to care and reintegration services is contingent on how their military service ends and discharge categorizations. Veterans who separate from the military on suboptimal grounds (eg, do not receive an honorable discharge) face the compounding problems of traditional transition obstacles and the social stigma of receiving an unfavorable discharge. These less-than-honorable (LTH) discharge statuses include general discharge, other-than-honorable (OTH) discharge, punitive discharge (including bad conduct and dishonorable discharges), and separation due to medical needs, fit, or for the convenience of the government. Across these varied statuses, many of these veterans are not eligible for federal services to address underlying mental health conditions that may have contributed to the separation or resulted from combat.^7^

While there is a deep body of literature exploring the short- and long-run consequences of service on veteran well-being,^8^ much less is known about how the specific characteristics of a military separation impact long-term outcomes. Servicemembers may separate from the military voluntarily via their own decision or involuntarily at their commander's decision. We argue that the needs of these 2 populations vary widely and merit particular attention by policymakers and researchers assessing the lifelong impacts of military service and the role of social support services for veterans. Given the restricted access to veterans’ support services, these involuntarily discharged veterans are underserved during the military–civilian transition, and there are substantial costs transferred onto the veteran's community.

In this paper, we identify 3 key, yet understudied, factors in the transitions between enlistment, discharge, and reintegration. First, we describe the military's role in separation and the long-run impacts of different discharge types. We found that, while a negative discharge is in the organization's best interest, this comes at the cost of the separating servicemember's immediate and enduring well-being as well as that of their family and community. Second, veterans must independently navigate and understand the benefits and rights available to them to be their own best advocates. While there is a robust social safety net for honorably discharged veterans, numerous VA programs are underutilized or disincentivized through negative discharge processes, limiting the efficacy of post-separation supports. We argue that the health care and social services needs of this population are legitimate, regardless of the social stigma around their discharge. Last, the lifelong impact of an LTH service record creates compounding challenges beyond the denial of benefits—such as substantial social stigma and consequences for housing and employment—which spill over beyond the veteran to their family, support network, and community. These consequences are punitive, persistent, and often overwhelmingly inhibit successful social reintegration. We argue in favor of offering a path forward from an LTH discharge to help these veterans regain some of their lost benefits and support their new civilian life.

## Type of military separation impacts transition support

Enlisted servicemembers enter the military through a signed contract specifying their service length. As servicemembers approach the end of their contract, they declare their intention to either re-enlist (eg, by signing another contract) or voluntarily separate from military service. Involuntary separation occurs when the military ends the servicemember's contract early. Typically, involuntary separations are performance- or event-based and fall into 2 categories: administrative and punitive. Administrative discharges are service-based determinations that include honorable, general, and OTH categorizations .^9,10^ Punitive discharges vary highly but broadly result from a court-martial trial and include willful and persistent misconduct, general felonies (eg, drunk driving, disorderly conduct), and more extreme offenses (eg, sexual assault, manslaughter, and arson). Approximately 16% of servicemembers receive LTH involuntary separations (10% General, 5% Other Than Honorable, and 1% Punitive).^11^

Servicemembers rely on formal transition programs as they move from active duty to veteran status, but these programs are not available across all separation types. In the event of voluntary separation, the servicemember enters a deliberate and established Department of Defense (DoD) Transition Assistance Program (TAP). The TAP consists of individual counseling sessions that start 1 year before separation. During these counseling sessions, servicemembers develop individualized plans for transitioning from the service. These individualized plans are then expanded through a series of classes, including financial planning, benefits, education, spousal support, and vocational and employment opportunities.^12,13^ Additionally, representatives from the VA, Department of Labor, and Small Business Administration hold multiple briefings and touchpoints during this final year to support the transitioning servicemember in accessing benefits and employment opportunities after separation.^13^ Over the years, TAP has received considerable attention and growth. With congressional support, numerous employment, educational, and housing programs have been created to supplement the military transition process.^14^ This preparatory year before the servicemembers’ transition provides a necessary structure and exposure to the experts and benefits while creating a collective approach towards overcoming transitional challenges. However, even with the structural support of TAP, the majority of veterans self-report struggling with the transition to civilian life.^5^

Involuntary separation timelines do not allow for participation in TAP. Often, these separations are event-based, rapidly executed, and result in LTH discharge. While determining the merits of an involuntary separation, the commander balances the rehabilitation potential for the servicemember against the impact on a unit's good order and discipline. While commanding officers may have discretion to begin moving a servicemember through TAP on an LTH discharge, not only is this not required but, in the event of fast separations, the servicemember would structurally not have access to a year of TAP support. As an example, once all rehabilitative efforts have been exhausted, separation goals are established to be between 15 and 50 working days for servicemembers in the Army, and shorter processing times are explicitly encouraged.^15^ While rehabilitative options are abundantly available at each military installation at the commander's discretion, the expedited separation timeline prioritizes military operations and functions over the separating servicemember's community reintegration.

The nature of these event-based administrative separations is unpredictable and involuntary.^7^ However, this does not prevent the military from enacting an adaptable transition plan to provide better support for these servicemembers as they move back into civilian life. The military arguably provides the most comprehensive benefits package of any US employer, including health, dental, housing, childcare, behavioral health, family advocacy, and food. As a result, servicemembers who are separated outside of their contracted service timeline face an abrupt loss of these exhaustive benefits, with limited (if any) transition. Coupled with an LTH discharge, reintegration can become overwhelming, placing this population at a disadvantage for a successful military–civilian transition^16^ and increasing the risk for behavioral health problems and criminal-legal involvement. This area merits further exploration of how the military can soften the landing for servicemembers who have fallen through the traditional military career path.

## Access to the Veterans Health Administration varies

Over the past decades, there has been a positive culture shift in recognizing the need for exhaustive and substantial veteran care. Compared with the experience of servicemembers separating during the Vietnam War, current veterans have access to a wide array of services and benefits through the VA, many of which were established as enlistment incentives during the transition to an all-volunteer service.^3,17^ Benefits include health care, education, training, life insurance, disability, home loans, burials, and pensions, all of which serve to support (eligible) veterans in their transition to civilian life.

Veterans become eligible for VA health care (administered via the Veterans Health Administration) conditional on 2 years of service and an honorable discharge, or in cases (such as in the event of disability or military sexual assault) where qualifying events led to separation.^3,18^ Honorably discharged veterans must only expend minimal effort to utilize their VA benefits.^2^ The VA assigns veterans to priority groups based on their income (determining relevant cost-sharing), service history, disability status, and eligibility for other benefits.^19^

Servicemembers with an OTH discharge can receive VA care for service-related disabilities, but not broader health concerns without a discharge upgrade. Servicemembers who receive punitive discharges are ineligible for VA health care unless the veteran successfully pursues a discharge upgrade or a Character of Discharge review.^18,20^ In these cases, the VA adjudicates the benefits received in LTH discharges on a case-by-case basis.^21^ Involuntarily separated servicemembers therefore solely bear the burden of accessing and understanding their available benefits. Servicemembers subject to other administrative discharges face a web of conditional and discretionary allowances that are buried in legal statutes and VA-coded regulations.^22^ For veterans who separate and are coping with reintegration, posttraumatic stress disorder (PTSD), limited benefits, and discharge status stigma, these barriers may prove insurmountable. Notable efforts are being made to improve accessibility for these veterans. The VA now allows limited care and immediate treatment options for veterans while they review the servicemembers’ applications for eligibility.^20^ However, these efforts do not address the baseline challenge of catchment into VA services for involuntarily separated servicemembers. How can this engagement be improved for the transitioning servicemember? Moreover, it is concerning that servicemembers may be disincentivized to seek or denied access to care because of their discharge status. This displaces the burden of support to veterans’ families and communities rather than acknowledging that, despite an LTH service record, these veterans’ need for care is legitimate.^23^

## LTH discharge may contribute to a cycle of misconduct

Veterans face disproportionate rates of criminal-legal system involvement, housing insecurity, and homelessness.^24^ A 2022 point-in-time count found that 33 129 veterans were experiencing sheltered or unsheltered homelessness.^25^ In 2016, over 107 000 veterans were housed in federal or state prisons, which does not even account for those held in jail custody.^26^ However, these estimates do not distinguish between discharge status, meaning that there is a substantial knowledge gap in how LTH discharge correlates with these kinds of adverse post-separation outcomes. We argue that discharge status creates specific structural barriers that increase the likelihood of criminal-legal involvement and housing insecurity.

An LTH discharge imposes lifelong social burdens on the servicemember, such as adverse employment background checks, housing biases from landlords, and the social stigma of having served “dishonorably.”^7^ Much like standard employment, the terms of involuntary separation may yield substantial resentment toward the military and associated service providers, such as the VA, impacting a veteran's willingness to engage with available services. Service-related behavioral health problems^11^ may go untreated once the servicemember loses access to VA benefits, which may impact the veteran's living situation and housing security. Further, post-separation, the veteran faces substantial barriers in establishing employment, health care, housing, and the other necessary factors of a civilian life.^27^ Research has found evidence of this increase in psychosocial problems, drug and alcohol use disorder, and criminal-legal system involvement among veterans with an LTH discharge relative to those discharged honorably.^28^

Recent policy changes have sought to recognize the unique needs of veterans in the criminal-legal system. Veteran Treatment Courts (VTCs) divert veterans from traditional criminal courts where they can receive access to specialized resources, opportunities to break the misconduct cycle, and support in their recovery.^29,30^ Firsthand accounts of VTC trials show that veteran defendants demonstrate a unique pride in their military service and a desire to seek help and regain a sense of lost honor,^31^ signaling that veterans are motivated to overcome an unfavorable discharge. We argue that this motivation provides an opportunity for the VA to develop and expand opportunities to upgrade veterans’ discharge status.

## New VA policies offer potential paths forward

The VA has recently opened a limited review process for upgrading discharge categorizations. Eligibility is limited to conditions that existed during the service period, such as traumatic brain injury, PTSD, sexual assault or harassment during service, and discharge due to sexual orientation under “Don’t Ask; Don’t Tell.”^18^ Importantly, veterans must themselves apply rather than being proactively recruited for upgrade. While it is unclear how many of these applications have been adjudicated or approved, this is an encouraging policy change that opens the door for expanding the scope of eligibility for an upgraded discharge review.

The current review process does not allow for civilian behavior or treatment to be considered in determining upgraded discharge categorizations. However, veterans may participate in—or be compelled by the court or the DoD to complete—behavioral health care treatment, anger management, and other post-separation resources directly related to the cause of the veteran's discharge. By allowing for rehabilitation to count toward discharge categorization upgrades, DoD would create opportunities (and incentives) for servicemembers’ long-term growth and recovery because of the potential for restored access to veterans’ benefits lost due to discharge status.

Additionally, we argue for the expansion of TAP to veterans who separate on accelerated timelines under LTH terms. While traditional TAP occurs during the months leading up to a veteran's separation, for expediently separated veterans the VA may consider online modalities to offer similar education and training to veterans who have returned to civilian life. Further, VA health care should be automatically provided for service-related injuries and conditions regardless of discharge status. While military benefits may be used as an incentive to promote good behavior during service, such an expansion would parallel civilian workers' compensation protections for on-the-job injuries regardless of terms of employment separation.

Finally, policymakers should consider limits to public access to veterans’ DoD Form 214 (DD-214)—Certificate of Uniformed Service. This 1-page document, available from the National Archives under the Freedom of Information Act,^32^ is a veteran's “proof” of service and specifies discharge status. Military service verification is required to receive benefits and is available in background checks for housing and employment.^33^ State policies vary on whether employers and landlords can request copies for DD-214s, with only some explicitly prohibiting employers from discriminating against current or future employees based on their military service.^34^ Such prohibitions have precedent in state “Ban the Box” efforts preventing employers from accessing job applicants’ arrest histories.^35,36^ However, research has found evidence of adverse unintended consequences for marginalized applicants, particularly young Black men, where employers facing limited objective information instead engage in statistical discrimination.^36,37^ Proposals to prevent employers from accessing DD-214s should be carefully assessed to avoid these unintended consequences for already marginalized veterans.

## Implications for researchers

Policymakers, health care providers, and researchers have long studied the challenges (such as “shell shock” and “battle fatigue”) faced by re-entering veterans since World War II.^8^ This body of research on the wide-ranging impacts of combat (and non-combat service) has resulted in societal shifts that have expanded veteran care beyond only combat-related physical disabilities and growing recognition of the complexity of veterans’ care.^38^ Posttraumatic stress disorder has prevalence rates (6.1%–9.2%) comparable to asthma (8.4%) and diabetes (13.4%) among the general population,^39^ but studies of veterans have found twice that, with 11%–23% of veterans exhibiting PTSD symptoms.^40^ Further, researchers have identified linkages between PTSD and depression, suicide, drug and alcohol abuse, violence, and inhibited veteran social relationships, conditions that impact not only the veteran but the veteran's family, friends, and community more broadly.^41^

The combined body of research on veteran well-being attempts to provide a net to catch, support, and provide solutions for the unique challenges of life after service. Rapid military–civilian transition from an involuntary separation is a knowledge gap within this veteran support net. Research focused on this starting point may prove invaluable to addressing the negative downstream impacts of an LTH discharge. However, there is substantial selection in existing studies on veterans, since those studies often use data from VA health care or recruit participant veterans from the VA directly.^24,42^ Because veterans with LTH discharges are by default ineligible for VA care, these studies by construction have limited access to LTH separated veterans^3^ and therefore limited understanding of outcomes and difficulties for these veterans. We argue for targeted and intentional research on LTH discharges using sampling beyond VA data/users to understand the long-term outcomes of general, OTH, and punitive discharge and how the state can best serve these veterans.

## Discussion

An incredible amount of research and energy focuses on veteran care, demonstrating how complex and significant these issues are. However, the current systems in place work against veterans with LTH discharges who often have restricted access to veterans’ benefits, especially those benefits that may improve their post-discharge transition. Despite this, there is limited research on these veterans exit from the military. Once separated, the inaccessibility and information asymmetry of benefits available result in ineligibility for, or underutilization of, formal veterans’ service channels, relocating the burden of care onto other social infrastructures. Key organizations (VA, DoD) can soften the impacts of LTH discharges through policy and program change. Expedient exit (in advance of typical contracted separation) from the military is an under-researched area for reform, and we argue that researchers studying veteran populations should carefully consider how discharge status may impact lived experience and access to support services. This can be supported by expanding VA data collection to include outcomes for veterans with LTH discharges. Last, the enduring societal punishment of an unfavorable discharge should be further examined.

The creation and implementation of new policies across both military and civilian domains require continued monitoring and analysis. Most directly, VA and DoD policymakers should consider establishing a standardized, endorsed, and supported transition program for servicemembers receiving an involuntary administrative separation. The case-by-case and expedient nature of their separation fails to support their transition needs and transfers the cost onto communities that are ill-equipped to handle these veterans’ needs. Additionally, we recommend federal guidance on the appropriate limitations to the use of military records to minimize discriminatory practices.

## Supplementary Material

qxae021_Supplementary_Data
